# Editorial: Protein Solubility and Aggregation in Bacteria

**DOI:** 10.3389/fmicb.2016.01178

**Published:** 2016-07-29

**Authors:** Salvador Ventura

**Affiliations:** Departament de Bioquimica i Biologia Molecular, Institut de Biotecnologia i Biomedicina, Universitat Autònoma de BarcelonaBarcelona, Spain

**Keywords:** bacteria, protein folding, protein aggregation, protein expression, functional amyloid, bacterial chaperones, prion-like proteins

For many years, the aggregation of proteins and polypeptides remained a neglected area of protein chemistry. It was only with the discovery that the insoluble deposits found in the organs and tissues of patients suffering from different diseases were enriched in a single, but different polypeptide, that the interest in understanding how and why these proteins aggregate arose (Fernàndez-Busquets et al., 2008). From that time on, the study of protein aggregation has evolved to become a key research topic, whose implications span disciplines like biochemistry, biomedicine, biotechnology, and nanotechnology.

The formation of insoluble protein aggregates is linked to more than 40 human diseases (Invernizzi et al., 2012). In all these disorders, the aggregated proteins assemble into a common β-sheet enriched supra-molecular structure, known as amyloid. A large number of higher eukaryotic biochemical pathways, from DNA replication to protein degradation, have been modeled first in prokaryotic organisms, providing important clues on the molecular basis of pathology (Dwyer et al., 2012). Scientists have long known that, very often, the heterologous expression of proteins in bacteria results in the accumulation of the target protein in insoluble deposits. However, only recently a number of laboratories have dared to exploit this well-characterized phenomenon to dissect the determinants of protein aggregation into amyloid assemblies (Villar-Pique and Ventura, 2012). Bacteria posses an intracellular environment that, while differing from that of eukaryotic cells, is physiologically more relevant than a test tube. Accordingly, they constitute privileged model systems to understand the mechanisms behind amyloid assembly and the cellular fitness cost associated with the formation of these aggregates (Navarro et al., 2014), but also to screen for effective modulators of amyloid aggregation with potential therapeutic applications (Villar-Piqué et al., 2012).

Protein aggregation constitutes a major bottleneck in the biotechnological production of protein based therapeutics. Consequently, a large effort has been devoted to the development of orthogonal strategies to increase soluble protein yields, including *in vitro* protein synthesis (Ventura, 2005). Bacterial extracts have become a convenient means to produce recombinant proteins *in vitro*, because they contain all the machinery required for protein synthesis. This technology has rendered a significant volume of data on the behavior of proteins during their cell-free synthesis. As illustrated by Tokmakov, these datasets can be exploited to derive the physicochemical and structural properties associated with the solubility and aggregation of eukaryotic proteins (Tokmakov). However, the cell is a very crowded environment and during cell-free experiments the concentration of cellular components is significantly diluted, questioning whether these assays recapitulate intracellular conditions. To address this question, the group of Taguchi has made an effort to synthetize more than 100 proteins in a bacterial cell-free system, either in the absence or in the presence of specific crowding agents. Their results demonstrate that the impact of crowding on aggregation is not generic, but protein-dependent (Niwa et al.).

Protein aggregates are not distributed homogeneously in the cytoplasm of bacteria, instead, they are mainly located at one or at the two poles of the organism. This localization has important functional consequences, from regulating signaling to protect the rest of the cell from misfolded species. In an original study, Emberly and co-workers from Simon Fraser University demonstrated that the localization of aggregating proteins in bacteria depends on their expression rates. This suggests that the localization of protein aggregates is constrained by aggregation itself, but also by nucleoid occlusion (Scheu et al.). When the accumulation of these polar aggregates results from the recombinant protein expression, they are named inclusion bodies (IBs). Despite they contain certain impurities, IBs are usually highly enriched in the target polypeptide. This property, together with their insolubility, allows for an easy purification of the recombinant polypeptide. In many cases, the protein can be recovered afterwards in its biologically active form upon *in vitro* unfolding and subsequent refolding, as demonstrated by the group of Panda for L-asparaginase (Upadhyay et al.).

Molecular chaperones, like trigger factor, the Dna KJE system and GroEL/GroES survey the protein quality in the bacterial cytosol. However, a dedicated mechanism is needed when proteins should be targeted to the bacterial membrane. Genevaux and co-workers from Université Paul Sabatier review the crucial role played by multitasking Sec B chaperones in this complex process (Sala et al.). Chaperones regulate proteostasis, but they also may allow the apparition of novel beneficial phenotypes. In this way, the Thomas Bentin's group demonstrates how GroEL/GroES over-expression increases cellular fitness and expands the mutational space. These effects provide an opportunity for bacteria to acquire tolerance, and even resistance, to antibiotics (Goltermann et al.).

Accumulating evidence indicates that different organisms exploit the special architecture of amyloid protein aggregates for functional purposes (Otzen, 2010). Functional bacterial amyloids constitute amazing macromolecular systems, where shifts in the folding and solubility of the embedded proteins in response to environmental factors critically affect activity, as reviewed by Boles and co-workers at University of Iowa (Syed and Boles). Two nice examples of the role played by these functional assemblies are provided in the works of Otzen's group and Lagos lab. In the first case, the authors described how amyloids in the *Pseudomonas* biofilm make a major contribution to the mechanical robustness of this extracellular matrix (Zeng et al.). In the second example, the authors identify the key residues accounting for the amyloid propensity of MccE492, a pore-forming bacteriocin whose antibacterial activity seems to be inactivated in the aggregated state (Aguilera et al.).

Prions are a special class of amyloids, in which the aggregated state becomes self-perpetuating. The prion phenomenon is best-known by its association with encephalopathies in mammals, but it also occurs in lower eukaryotic organisms, like yeast, where it is exploited for functional purposes. The self-assembly of yeast prions relies on the presence of long and intrinsically disordered glutamine/asparagine rich domains. These domains are both necessary and sufficient for self-templating protein aggregation. Giraldo and his group, showed that a fragment of these domains could be replaced by the protein sequence of RepA-WH1, a bacterial protein with amyloid-like properties, without losing the intracellular aggregation potential of the resulting chimera in yeast (Gasset-Rosa and Giraldo). This finding opens up the possibility that prion-like proteins would also exist in prokaryotes. Accordingly, the group of Ventura, using a previously developed computational approach (Espinosa Angarica et al., 2014), identified more than 2000 putative prion candidates in bacterial proteomes (Iglesias et al.). A significant number of these proteins are involved in DNA transcription and protein translation, therefore, playing a crucial role in the regulation of biochemical pathways. One outstanding example of this type of proteins is the Rho terminator factor. Ventura and co-workers demonstrate that in the pathogen *Clostridium Botulinum* this essential protein contains a prion-like domain, with the ability to self-assemble into amyloid structures similar to those found in yeast prions (Pallares et al.).

Overall, it is clear that the study of protein solubility and aggregation in bacteria is a highly dynamic field with the potential to provide very relevant insights and tools to understand and control deleterious and beneficial protein self-assembly.

## Author contributions

The author confirms being the sole contributor of this work and approved it for publication.

### Conflict of interest statement

The author declares that the research was conducted in the absence of any commercial or financial relationships that could be construed as a potential conflict of interest.
